# The Effect of Preoperative Oral Melatonin on Postoperative Pain after Lumbar Disc Surgery: A Double-Blinded Randomized Clinical Trial

**DOI:** 10.4314/ejhs.v32i6.17

**Published:** 2022-11

**Authors:** Afshin Gholipour Baradari, Mohammad Reza Habibi, Mohsen Aarabi, Samira Sobhani, Anahita Babaei, Amir Emami Zeydi, Faraz Ghayoumi

**Affiliations:** 1 Department of Anesthesiology, Faculty of Medicine, Mazandaran University of Medical Sciences, Sari, Iran; 2 Department of Epidemiology, Faculty of Medicine, Mazandaran University of Medical Sciences, Sari, Iran; 3 Department of Medical-Surgical Nursing, Nasibeh School of Nursing and Midwifery, Mazandaran University of Medical Sciences, Sari, Iran; 4 Faculty of Pharmacy, Tehran University of Medical Sciences, Tehran, Iran

**Keywords:** Melatonin, Acute Pain, Laminectomy, Pain, Postoperative

## Abstract

**Background:**

Despite advances in surgical and anesthesiology techniques, many patients continue to experience postoperative pain after lumbar disc surgeries. The aim of this study was to investigate the effect of preoperative oral melatonin on the severity of postoperative pain after lumbar laminectomy/discectomy.

**Methods:**

In this double blinded randomized controlled clinical trial 80 patients undergoing an elective mini-open microdiscectomy surgery at Imam Khomeini educational hospital in Sari, Iran, were selected and randomly assigned into one of four groups. Patients in group A, B, C, and D received 3, 5 and 10 mg melatonin or placebo tablets one hour before surgery, respectively. Using the visual analogue scale (VAS) the severity of pain, nausea and vomiting, pruritus, and use of narcotics were assessed immediately after surgery and before leaving the post-anesthesia care unit, 6, 12 and 24 hours postoperatively.

**Results:**

In all three groups receiving melatonin at all three different doses, postoperative pain was significantly less than the placebo group (P<0.01). There were no statistically significant differences in postoperative pain level between the three groups receiving melatonin (P>0.05). The amount of opioid received by the patients within 24 hours after surgery had statistically significant differences within the groups (P=0.043, F=2.58). The results of post hoc analysis in terms of postoperative pain intensity showed statistically significant differences between the two groups receiving melatonin at a dose of 5 mg and the placebo group (P=0.04). No serious side effects reported in four groups.

**Conclusion:**

The use of oral melatonin with a dose of 5 mg, 1 hour before the surgery as an inexpensive method can effectively reduce pain intensity as well as the amount opioid use after lumbar laminectomy and discectomy.

## Introduction

Lumbar discectomy is the most commonly performed surgical procedure for the treatment of patients with lumbar radiculopathy caused by for lumbosacral disc herniation (1). Despite attempts to increase the public awareness to pain assessment programs and the development of standardized pain management pathways for postoperative pain, many patients still experience severe pain following their surgery (1, 2–3). Patients undergoing decompressive procedures of spine such as discectomy, could potentially experience moderate to severe postoperative pain (1–2).

Postoperative pain can cause adverse physiological effects such as inadequate depth of breathing and inadequate discharge of respiratory secretions, atelectasis and pulmonary complications, increased heart rate and blood pressure, ileus and prolonged bed rest. Immobility secondary to pain could potentially lead to an increase in incidence of deep vein thrombosis (4–5). In addition, in patients undergoing surgery on the spinal vertebral column, postoperative pain can postpone the onset of walking and physiotherapy, increase hospital stay and cost of surgery and alter the patient's sense of recovery and even adversely affect surgical outcomes (6).

Therefore, utilizing pain reduction pathways with low rate of complications is an important postoperative goal. Narcotic drugs especially in an injectable form are the first line pain relief modality and are widely used to relieve acute postoperative pain following lumbar spine surgery (5, 7–8). On the other hand, pain as a multifactorial phenomenon cannot be completely eliminated using single-drug treatment interventions and narcotic drugs. Use of multimodal pain management as an approach to post-operative pain control has been well documented. In addition, the use of narcotics is associated with dose-related complications such as respiratory depression, nausea, vomiting, urinary retention, itching, drowsiness or postoperative ileus (9). A newer approach has been well established with use of pre-emptive analgesia to reduce postoperative pain and narcotic consumption (10).

One of the most commonly used drugs for the reduction of acute pain and improvement of analgesic effects after the surgery is melatonin. Melatonin or n-acetyl methoxy-tryptamine is a hormone secreted by the pineal gland in the brain. Melatonin has important biological effects in the body and plays an important role in adjusting the sleep cycle and awakening process (11–12). Studies have shown the protective role of melatonin in various diseases such as cancer, cardiovascular diseases, Alzheimer, diabetes, mood disorders, digestive diseases, fibromyalgia and psychiatric disorders (12). Studies on patients undergoing surgical interventions have shown that surgery and anesthesia have been associated with a reduction of plasma levels of melatonin. Therefore, supplemental use of melatonin in patients undergoing surgery is recommended (13–14). Additionally, some studies have shown the positive effects of melatonin during anesthesia and surgery including a reduction of preoperative anxiety, postoperative delirium, need for anesthetic drugs and pain intensity (15–16).

The analgesic effects of melatonin on the improvement and enhancement of morphine antinociceptive have been shown in various animal studies (16–17). While the precise mechanism of the analgesic effects of melatonin is not fully understood, the stimulation of β-endorphin secretion as well as its effect on various receptors including opioid, benzodiazepines, muscarinic, serotonergic receptors in the posterior horn of the spinal cord, as well as the central nervous system is suggested (15, 18). Controversial results have been reported in various clinical studies on the postoperative analgesic effects of melatonin in different doses (including 3, 5, 6 and 10 mg) (19–24).

Therefore, given that melatonin is a safe drug, more clinical trials are required to evaluate the analgesic effects of melatonin and compare its different doses for understanding the most effective and appropriate dosage for administration (15–16). In addition, very few studies have been done to investigate the effects of melatonin in patients undergoing lumbar discectomy. Therefore, given the potential of melatonin as a safe drug to decrease postoperative pain, this study was conducted to investigate the effect of preoperative oral melatonin on the severity of pain following lumbar laminectomy discectomy. We hypothesized that using oral melatonin in preoperative period could provide additional pain relief after lumbar laminectomy/discectomy.

## Methods

This study was a parallel, double blinded randomized controlled clinical that was carried out between April 2016 and January 2017. After obtaining approval from the Ethics Committee of the University and informed consent from patients, 80 patients who were undergoing open lumbar spine laminectomy and discectomy at one or two levels at Imam Khomeini Educational Hospital in Sari, Iran, were recruited. They were classified by the American Society Anesthesiology (ASA) as class I and II of anesthesia and were admitted to a teaching hospital in an urban area of Iran.

Inclusion criteria were age 35–70 years, confirmation of the diagnosis using physical examination, computed tomography (CT) scan and magnetic resonance imaging (MRI) and undergoing an elective discectomy surgery. Exclusion criteria were unwillingness at any time to continue participation in this study, emergency discectomy surgery, involvement of more than two lumbar discs, opiate drug use up to 12 hours before the intervention, alcohol or drug abuse and occurrence of any unusual complications during the surgery. Also, patients with the history of prior spinal surgery and known allergy to the drugs used in the study were excluded.

Eligible patients were randomly assigned into four equal sized groups, using simple randomization technique, as A, B, C and D. For ensuring allocation concealment, the sequentially numbered, opaque, sealed envelope technique was used by a nurse who was unaware of the study groups. Before the surgery, the patients were provided with adequate explanations and education on how to report pain severity, nausea, vomiting and itchiness after the surgery using the Visual Analog Scale (VAS).

One hour prior to surgery an anesthesiology nurse helped administer group A and B one 3mg and 5mg melatonin tablet (Nature Made^®^, USA), respectively. Groups C and D received one 10mg tablet of melatonin and placebo (which was the same shape of melatonin) one hour before their surgery, respectively. In the operating room, all patients were placed under general anesthesia using a similar anesthetic protocol including midazolam (0.1mg/kg), fentanyl (2µ/kg), neodonal (5 mg/kg), atracorium (0.5 mg/kg), 50% N2O, isoflore MAC 0.6–1 and morphine 0.1mg/kg and atracurium based on the patient need 0.01 mg/kg. All surgeries were performed by one surgeon via a same approach.

After the surgery, pain was assessed using the VAS and morphine 5 mg was administered to those patients with pain severity more than 3. Pain severity, nausea, itching and vomiting in the groups were evaluated and documented after the surgery and before leaving the recovery room and at 6, 12 and 24 hours postoperatively. The evaluation of the above variables was performed by a collaborator nurse who was blinded to the groups and received sufficient education about the study process. In addition, the amount of fentanyl consumed during anesthesia was also 50 mg per hour.

The primary outcome was the severity of postoperative pain and secondary outcomes were opioid use, nausea, vomiting, pruritis and patient satisfaction. To control postoperative pain, all patients received paracetamol (Apotel®) 1 g every 8 hours. Patients did not received any antiemetic prophylaxis. At the beginning of the study, weight and height of the patients in kg and cm were measured, respectively. Body mass index (BMI) of the patients was also calculated using standard methods. This information was documented in the relevant sheet with specifications such as age, level of education, duration of surgery, duration of anesthesia, lumbar disc level involved, patient's first request to receive pain medication after surgery and the amount of opioid used within 24 hours of the surgery.

**Sample size**: A priori sample size were calculated using GPower3.1 with the formula for calculation of samples of repeated measures, based on a presumed effect size of 0.3, a statistical power of 80%, and a type I error of 5%. The overall proper sample size was found to be 74 participants. We therefore recruited 80 patients to account for any dropouts.

**Statistical analysis**: After data collection, statistical analysis was performed using descriptive and inferential statistics via the SPSS v.18 software. Shapiro-Wilk test was used to assess normality of the data. Chi-Square/Fisher exact test and t-test were used for qualitative and quantitative variables, respectively. Repeated Measure Analysis of Variance (ANOVA) or Kruskal-Wallis tests were used to evaluate pain severity, nausea and pruritus, following by a Tukey test as a post hoc analysis. P-value less than 0.05 was considered statistically significant.

**Data sharing**: All relevant data and methodological detail pertaining to this study are available to any interested researchers upon reasonable request to corresponding author.

## Results

**Figure 1:**
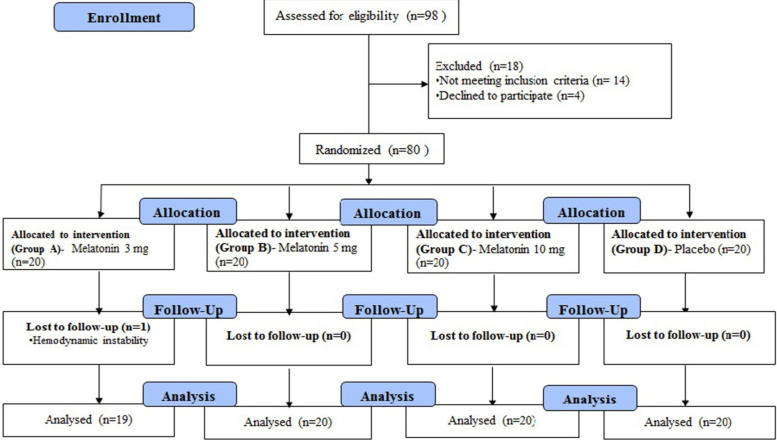
The process of the study according to the CONSORT flow diagram

In this study, 98 patients were evaluated, of which 80 patients were eligible to be included in this study and assigned into the groups. Except for one patient in group A, all other patients completed the study, and data from all these patients were analyzed (Figure 1). The demographic and clinical characteristics of the patients in the groups are shown in Table 1. No statistically significant differences between the groups in terms of age, gender, BMI, level of education, place of residence, duration of surgery and anesthesia, the amount of fentanyl and lumbar disc level involved were reported (Table 1).

**Table 1:** The demographic characteristics of the patients in the groups

Variable	Groups				P-value

	Group A (n=19)	Group B (n=20)	Group C (n=20)	Group D (n=20)	
Age (year)	37.05±4.7	36.8±6.2	40.3±8.1	39.2±7.9	0.32^a^
Mean (SD)					
Gender (male/female)	10/9	8/12	7/13	12/8	0.36^b^
BMI	26.75±2.6	26.34±2.4	27.02±4.1	28.82±4.6	0.15 a
Mean (SD)					
Education level					
Illiterate	0(0)	2(10)	4(20)	1(5)	0.23 b
Under diploma	7 (36.8)	6 (30)	7 (35)	11 (55)	
Diploma and higher	12 (63.2)	12 (60)	9 (45)	8 (40)	
Residence					
Rural	9 (47.4)	7 (35)	7 (35)	6 (30)	0.71 b
Urban	10 (52.6)	13 (65)	13 (65)	14 (70)	
Duration of surgery	127.63±28.9	123.75±25.2	140±32.9	131.25±29.4	0.34 a
Mean±SD					
Duration of	153.95±32.3	150.75±30.3	165.25±33.6	155.75±28.06	0.49 a
Anesthesia					
Mean±SD					
Amount of fentanyl	110.53±31.5	105±27.6	117.5±33.5	110±44.7	0.73^a^
(mg) Mean±SD					
Involved lumbar vertebra n(%)					
L4-L5	14 (73.7)	18 (90)	17 (85)	17 (85)	0.57 b
L5-S1	5 (26.3)	2 (10)	3 (15)	3 (15)	

a ANOVA test

b Chi-Square test

BMI: Body Mass Index

**Figure 2:**
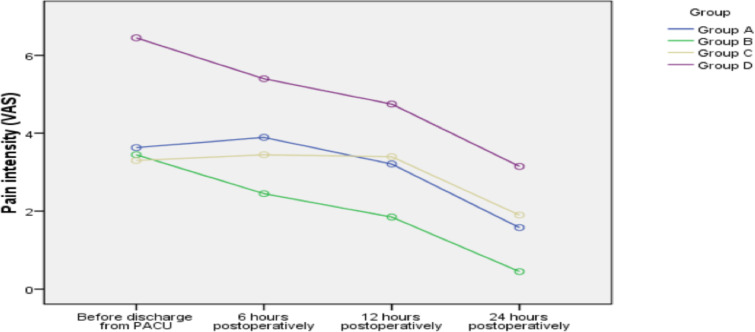
Changes in pain intensity in the groups during the follow up period

**Changes in pain intensity after the surgery**: depicted in Table 2 and Figure 2, the ANOVA test concluded that, irrespective of the groups, changes in pain intensity were statistically significant (time effect; P<0.001), but the changes in two groups were almost similar (interaction effect; P= 0.24). In addition, there were statistically significant differences between the groups in terms of pain intensity regardless of time (group effect; P<0.001). Tukey's post hoc test for the assessment of differences between the groups showed that pain intensity in all three groups receiving melatonin at different doses was significantly less than the placebo group (P<0.001). Also, there were no statistically significant differences between the three groups receiving melatonin in different doses in terms of postoperative pain intensity (P>0.05).

**Table 2:** Changes in pain intensity and nausea in four groups during the follow up period

Variable		Time				P-value		
		
		T1	T2	T3	T4	Time effect	Group effect	Time*group effect (interaction)
**Pain**	Group A	3.63±2.5	3.89±1.91	3.21±1.7	1.58±1.6	<0.001	<0.001	0.24
**intensity**	Group B	3.45±1.7	2.45±0.9	1.85±1.2	0.45±0.7			
	Group C	3.30±2.6	3.45±1.9	3.41±2.1	1.9±2.4			
	Group D	6.45±2.8	5.41±2	4.75±2.2	3.15±2.5			
**Nausea**	Group A	0.53±1.3	0.05±0.2	0	0	0.11	0.58	0.66
**intensity**	Group B	0.2±0.8	0.05±0.2	0	0.15±0.6			
	Group C	0.1±0.4	0.4±1.7	0	0.15±0.6			
	Group D	0.5±1.5	0.55±1.8	0	0.15±0.6			

**T1**: Before discharge from the post anesthesia care unit (PACU); **T2**: 6 hours after the surgery; **T3**: 12 hours after the surgery; **T4**: 24 hours after the surgery

**Figure 3:**
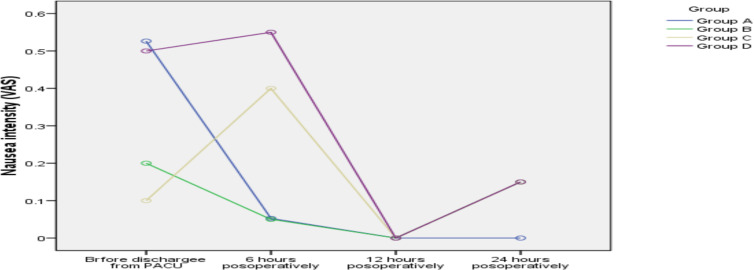
Changes in the severity of nausea in the groups during the follow up period.

**Changes in the incidence of postoperative nausea**: According to Table 2 and figure 3, the repeated measures ANOVA showed that, regardless of the groups, changes in the severity of nausea were not statistically significant (no time effect) (P=0.11). In addition, changes in the groups were almost similar (without interacting effects) (P=0.58). Also, no statistically significant differences were reported between the groups in terms of severity of nausea, regardless of time (no effect of the group) (P=0.66).

**The amount of requested and received opioids within 24 hours of the surgery:** According to Table 3, there were no statistically significant differences between the groups in terms of the number of patients receiving opioid drugs after surgery (P=0.1). The ANOVA test showed statistically significant differences in the amount of opioid received within 24 hours between the groups (Table 3) (P=0.043, F=2.58). To determine the difference between the groups, the Tukey's post hoc test was used indicating a statistically significant difference between the two groups receiving melatonin at a dose of 5 mg (group B) and the placebo group (group D) (P=0.04).

**Table 3:** The requested and received opioids within 24 hours after the surgery

Variable	Groups				
		
	Group A (n=19)	Group B (n=20)	Group C (n=20)	Group D (n=20)	P-value
The amount of received opioids after the surgery Mean±SD	3.95±8.5	2.5±7.6	4±9.8	11±13.3	0.043
Request by the patients for opioids after the surgery N (%)					
Yes	4 (21.1)	3 (15)	7 (35)	10 (50)	0.1
No	15 (78.9)	17 (85)	13 (65)	10 (50)	

**Changes in the incidence of pruritus and vomiting after the surgery**: None of the patients experienced postoperative pruritus. In terms of the incidence of vomiting, 2 patients in group A and 1 patient in group B and 2 patients in the placebo group had postoperative vomiting which were not statistically significant. (P=0.641). We did not observe any statistically significant differences between the groups in incidence of postoperative vomiting (P=0.524). There were no other complications in all four groups.

## Discussion

The findings of this study showed that preoperative oral melatonin administration significantly reduced the severity of postoperative pain compared with placebo in patients undergoing one or two levels lumbar open laminectomy and discectomy. However, no statistically significant dose dependent differences were reported between the three groups receiving melatonin in regards to postoperative pain intensity. In addition, postoperative opioid administration in all three melatonin groups was less than the placebo group. However, this difference was statistically significant only between the two groups receiving melatonin at a dose of 5 mg and the placebo group.

A study revealed that using of 10 mg of melatonin before cesarean section significantly reduces the severity of patients' pain, increases the duration of postoperative analgesia, reduces the need for analgesics after surgery and resumption of physical activity, without any major side effects (2).

Caumo et al. showed that taking 5 mg melatonin tablets one night and 1 hour before the surgery in patients undergoing abdominal hysterectomy caused a significant reduction in pain intensity and anxiety in the first 24 hours after the surgery compared with the control group. In addition, patients receiving melatonin had significantly less need to take morphine in the postoperative period (19), which was consistent with the findings of this study. Another study on patients undergoing abdominal hysterectomy showed that oral administration of melatonin (5 mg) and clonidine (100 µg) one night before and 1 hour before the surgery had similar effects on reducing pain intensity, anxiety and the use of morphine after the surgery. However, these effects in these two groups were greater than the placebo group (22).

Also, the positive effect of melatonin 10 mg as a premedication on the pain of tourniquet and the improvement of pain control in patients undergoing elective surgery has been reported (21). Another study assessed the effect of preoperative melatonin on sedation, sleep quality and postoperative pain in patients undergoing elective prostatectomy. It showed that taking oral melatonin 6 mg on the night before the surgery and one hour prior to the surgery significantly decreased pain intensity, fentanyl consumption during surgery and postoperative tramadol in patients receiving melatonin compared to the control group (24). Experimental studies have evaluated the frequency of analgesic use, antihyperalgesic, anti-inflammatory and antiallodynic effects of exogenous melatonin indicating the dose-related effects of exogenous melatonin (17, 25–27).

The precise mechanism of the analgesic effect of melatonin has not been accurately recognized. However, possible analgesic effects of melatonin are the role of β-endorphins, GABA and opioid receptors (1) and Nitric oxide arginine pathways (28). Melatonin has been shown to increase the release of endorphins from the pituitary gland. Naloxone blocks the binding of betaendorphins to opioid receptors and antagonizes the analgesic effects of melatonin (17–18) In addition, melatonin may interact with opioidergic receptors, benzodiazepine, muscarinic, nicotine, serotonergic, and α1 and α2 adrenergic receptors, and most importantly MT1/MT2 melatoninergic receptors located on the posterior horn of the spinal cord and the central nervous system to create antianalgesic effects (29). Since the long-term analgesic effects of melatonin can be blocked by naloxone, opioid receptors play a role in melatonin analgesic activities (30). Melatonin also seems to reduce pain, especially pain due to inflammation by reducing the production of nitric oxide, which plays an important role in modulating and directing pain-related information (31).

Following early studies on melatonin's analgesic effects, it has been shown that the circadian rhythm plays a role in the feeling of pain (32). Animal studies have shown that in the darkness, when melatonin is at its highest level, animals have the least sensitivity to pain and the highest sensitivity to morphine (33–34). Administration of melatonin can improve sleep and reduce anxiety, thereby reduce the severity of pain (27). A positive relationship between anxiety and pain in clinical settings is recognized. It has been shown that anxiety due to pain can increase the perceived pain in patients (35–36).

Another study compared the effect of melatonin and midazolam, as a premedication, in patients undergoing laparoscopic cholecystectomy using general anesthesia. Patients were randomly assigned into three groups: (i) patients receiving melatonin tablets 3 mg a night before the surgery; (ii) patients receiving midazolam tablets 3.75 mg; (iii) patients receiving placebo tablets. It was shown that in the postoperative period, the anxiety level in all groups was less in the melatonin group than in the placebo group (20).

In the present study, administration of melatonin tablets with all three doses of 3, 5 and 10 mg caused significant changes in the severity of postoperative pain compared with the placebo group. However, in the three groups receiving melatonin with different doses, no statistically significant differences in the severity of postoperative pain were reported. In terms of postoperative opioid use, only the group receiving 5mg of melatonin (Group B) reached statistical significant difference compared to the placebo group. However, the other two groups receiving melatonin (Group A and C) also received less opioids postoperatively compared with the placebo group, albeit not statistically significant. In addition, no significant differences in the incidence of vomiting and severity of nausea and vomiting between the groups were found. No other side effects in any of the groups were reported.

It seems that the use of melatonin at a dose of 5 mg before the surgery is an effective and safe option for reduction of postoperative pain intensity, amount of postoperative opioid consumption and higher patients' satisfaction. Experimental studies on animals showed the antinociceptive effects of melatonin in doses ranging from 0.1 mg/kg to 300 mg/kg (25). In a study on 61 healthy patients who received up to a maximum of 20 mg sublingual melatonin, the dose-dependent effects of melatonin for pain relief and the serum levels of melatonin were found; however, the total serum levels of melatonin were in the normal range (37). Different clinical studies have shown the analgesic effect of oral melatonin with a dose 10-3 mg (38). While animal and clinical studies have shown dose-dependent antinociceptive effects of melatonin, no decisive conclusion can be drawn regarding the optimal dose of melatonin in terms of its analgesic effects (39). Therefore, it may be recommended to administer melatonin 5–20 mg to create its analgesic effects (37–39), which should be studied in the future.

No major side effects have been reported in regards to administration of melatonin by patients. Melatonin has been used for over five decades both clinically and in various research projects and no significant side effects have been reported except for mild side effects such as drowsiness, dizziness, nausea and headache. More studies have shown that these effects are somewhat similar to placebo treatment (39). In addition, a systematic review examined the effects and safety of melatonin during surgery indicating that the use of melatonin was safe and had no significant side effects (40).

There are some limitations in the present study that need to be addressed. Our study is a single-center clinical trial, and selective bias is possible. Pain score is subjective and several factors can affect the severity of pain. Therefore, this should be mentioned as another limitation of present study. In conclusion, our study showed that using 5mg of oral melatonin 1 hour before one or two level lumbar spine laminectomy/discectomy is an inexpensive and safe method to effectively and efficiently reduce postoperative pain intensity and opioid use in these patients.
